# Dengue Incidence and *Aedes* Vector Collections in Relation to COVID-19 Population Mobility Restrictions

**DOI:** 10.3390/tropicalmed7100287

**Published:** 2022-10-07

**Authors:** Sinnathamby Noble Surendran, Ratnarajah Nagulan, Annathurai Tharsan, Kokila Sivabalakrishnan, Ranjan Ramasamy

**Affiliations:** 1 Department of Zoology, University of Jaffna, Jaffna 40000, Sri Lanka; 2 Faculty of Applied Science, University of Vavuniya, Vavuniya 43000, Sri Lanka

**Keywords:** *Aedes aegypti*, *Aedes albopictus*, COVID-19, dengue incidence, dengue vectors, dengue transmission, mosquito vector–human interactions, population movement restrictions, Jaffna, Sri Lanka

## Abstract

Contrary to expectation, dengue incidence decreased in many countries during the period when stringent population movement restrictions were imposed to combat COVID-19. Using a seasonal autoregressive integrated moving average model, we previously reported a 74% reduction in the predicted number of dengue cases from March 2020 to April 2021 in the whole of Sri Lanka, with reductions occurring in all 25 districts in the country. The reduction in dengue incidence was accompanied by an 87% reduction in larval collections of *Aedes* vectors in the northern city of Jaffna. It was proposed that movement restrictions led to reduced human contact and blood feeding by *Aedes* vectors, accompanied by decreased oviposition and vector densities, which were responsible for diminished dengue transmission. These findings are extended in the present study by investigating the relationship between dengue incidence, population movement restrictions, and vector larval collections between May 2021 and July 2022, when movement restrictions began to be lifted, with their complete removal in November 2021. The new findings further support our previous proposal that population movement restrictions imposed during the COVID-19 pandemic reduced dengue transmission primarily by influencing human–*Aedes* vector interaction dynamics.

## 1. Introduction

**Figure 1:**
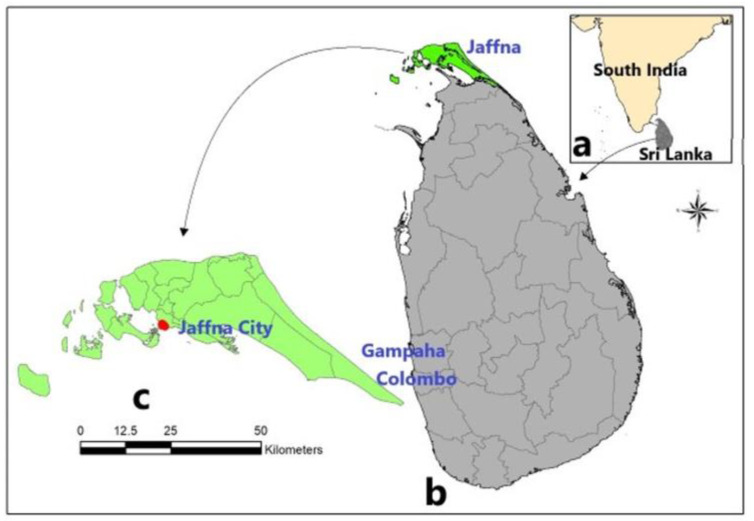
Map showing (**a**) the location of Sri Lanka in relation to India; (**b**) the boundaries of the 25 districts including Colombo, Gampaha, and Jaffna districts that have high dengue incidence; and (**c**) the location of the city of Jaffna within the Jaffna district.

The COVID-19 pandemic, by diverting health resources and reducing mosquito vector control programs, had been expected to increase the prevalence of dengue and other mosquito-borne diseases in endemic countries. There was instead a marked decrease in dengue incidence during the most intense phase of the pandemic in Sri Lanka [1,2,3,4,5] and many other countries [6]. COVID-19 was first detected in Sri Lanka in January 2020, and drastic public health measures to contain its transmission were initiated on 17 March 2020 [1]. A 74% reduction in total dengue cases in the whole country occurred during the period from 1 March 2020 to 30 April 2021 compared with the number of cases predicted based on the dengue incidence in the five-year period immediately preceding the pandemic (January 2015 to February 2020) [1]. The decrease in dengue incidence during the period from 1 March 2020 to 30 April 2021 occurred in all 25 administrative districts of Sri Lanka, with a reduction of 89% in the northern Jaffna district (Figure 1) [1]. All four dengue virus serotypes, DENV 1–4, are presently found in Sri Lanka, with regional and temporal variation in their prevalence [7].

The severe restrictions on movement of people that were in place from 1 March 2020 to 30 April 2021 in Sri Lanka [1] (period A) in an effort to reduce COVID-19 transmission were variably eased and reimposed from 1 May 2021 until 22 November 2021 (period B) but removed altogether, with the full opening of all schools, from 22 November 2021 onward (period C). Measures that restricted population mobility during the three periods are summarized in Table 1, together with the range of the Government Stringency Index, which is an internationally developed composite measure of nine COVID-19 response matrices [8]. Vaccination and a reduction in the number of severe COVID-19 cases were important factors that allowed for the removal of population movement restrictions that had closed schools and offices and, at times, restricted people to their residences (Table 1). 

*Aedes aegypti* and *Ae. albopictus* are the primary and secondary vectors, respectively, of dengue worldwide [9] and in Sri Lanka [1,10,11]. Ovitrap collections in the Gurunagar municipal ward of Jaffna city (Figure 1) showed that the monthly numbers of *Ae. aegypti* and *Ae. albopictus* larvae collected in the period August 2020 to April 2021 were significantly reduced by 88.6%, with a significantly lower proportion of *Ae. aegypti* than *Ae. albopictus*, compared with the pre-pandemic period of March 2019 to December 2019 [1]. *Aedes* vector densities and dengue cases rapidly increase with the onset of monsoonal rains in Sri Lanka, predominantly during the northeast monsoon season from October to December in the Jaffna district [1,10,11]. We previously proposed that restricted population movement during the height of the COVID-19 pandemic reduced human biting rates of *Aedes* vectors and egg laying, and consequently dengue transmission and incidence in Sri Lanka [1]. We now show that data on dengue incidence in Sri Lanka during the period from 1 May 2021 to 31 July 2022, a period during which there was a phased relaxation of movement restrictions, followed by their complete removal on 22 November 2021 onward (Table S1), as well as *Aedes* ovitrap collections in Gurunagar, are consistent with this proposal. 

## 2. Materials and Methods

### 2.1. Diagnosis and Reporting of Dengue in Sri Lanka

A diagnosis of dengue must be reported, by law, to the Ministry of Health of Sri Lanka. Dengue and dengue haemorrhagic fever are always considered possible in patients presenting with an acute onset of fever accompanied by (i) headache (especially retro-orbital pain), (ii) myalgia/arthralgia, (iii) rash (diffuse, erythematous, macular), (iv) haemorrhagic manifestations (petechiae, positive tourniquet test, etc.), (v) leukopenia (<5000/mm^3^), (vi) rise in haematocrit of 5–10%, and (vi) thrombocytopenia (≤150,000/mm^3^) [12]. Acute fever with thrombocytopenia and at least two of the other clinical manifestations are considered sufficient for a diagnosis of dengue according to local guidelines [12]. Lateral flow tests for detection of dengue virus NS1 antigens are only performed if test kits are available. 

### 2.2. Data on Dengue Incidence and Rainfall

Monthly data on dengue cases from 1 January 2015 until 31 July 2022 were obtained from the Government of Sri Lanka Epidemiology Unit website [5], and monthly rainfall data for Jaffna district from January 2015 to July 2022 were obtained from the Government of Sri Lanka Meteorology Department. 

### 2.3. Prediction from Pre-Pandemic Data of the Expected Number of Monthly Dengue Cases from 1 May 2021 to 31 July 2022

An extension of the widely used Autoregressive Integrated Moving Average Model for time series data forecasting that supports the direct modelling of the seasonal component of the series, termed the Seasonal Autoregressive Integrated Moving Average Model (SARIMA), was utilized as previously described [1]. The SARIMA model takes overall trends, as well as seasonal changes in dengue incidence, into consideration [1,13]. Dengue incidence in Sri Lanka is principally associated with seasonal monsoon rainfall [1,10,11]. The anticipated numbers of monthly dengue cases from 1 May 2021 to 31 July 2022 for the whole country and each of the administrative districts were predicted using the SARIMA model based on the dengue case numbers for the corresponding months in the immediately preceding five-year pre-pandemic period from January 2015 to February 2020. 

### 2.4. Ovitrap Collections of Aedes Larvae in Gurunagar, Jaffna 

*Aedes* larval collections were performed in Gurunagar (9°39′12.6″ N, 80°01′03.5″ E), a coastal municipal ward in a built-up area of Jaffna city, from May 2021 to April 2022, as previously described in Gurunagar during 2019 [10] and 2020–21 [1]. Conventional black plastic ovitraps with water obtained from the nearest domestic water source (i.e., well or tap) with a 2 × 15-cm plywood paddle resting against the inside upper rim were used [1,10]. Ten ovitraps were placed at different locations in Gurunagar. Larvae were collected fortnightly from each ovitrap and maintained in a contained insectary of the Department of Zoology in the University of Jaffna; emerging adult mosquitoes were identified at the species level as previously described [1,10]. Ovitrap larval collections were not possible after April 2022 due to the chronic fuel shortage experienced throughout Sri Lanka. 

### 2.5. Statistical Analysis

The non-parametric Wilcoxon signed-rank test was used to determine the significance of differences between predicted and actual numbers of dengue cases for each month from 1 May 2021 to 31 July 2022 for each of the 25 districts and, as well as the whole of Sri Lanka. The chi-square test was used to determine the significance of differences in proportions of ovitraps that were positive for *Ae. aegypti* and *Ae. albopictus* during the period from 1 May 2021 to 30 April 2022. The non-parametric Mann–Whitney U test was used to compare the numbers of *Ae. aegypti* and *Ae. albopictus* collected from ovitraps during the same period. 

## 3. Results

### 3.1. Dengue Incidence from 1 May 2021 to 31 July 2022 in All of Sri Lanka

The nationwide predicted and actually reported number of dengue cases for each month during the 15-month period between 1 May 2021 and 31 July 2022 are shown in Supplementary Table S1. The predicted and reported total numbers of dengue cases in the country from 1 May 2021 to 31 July 2022 were 106,468 and 54,596 cases respectively. The reported cases represented a statistically significant (*p* =  0.00006) 49% reduction relative to the predicted number of cases for the entire 15-month period. 

**Figure 2:**
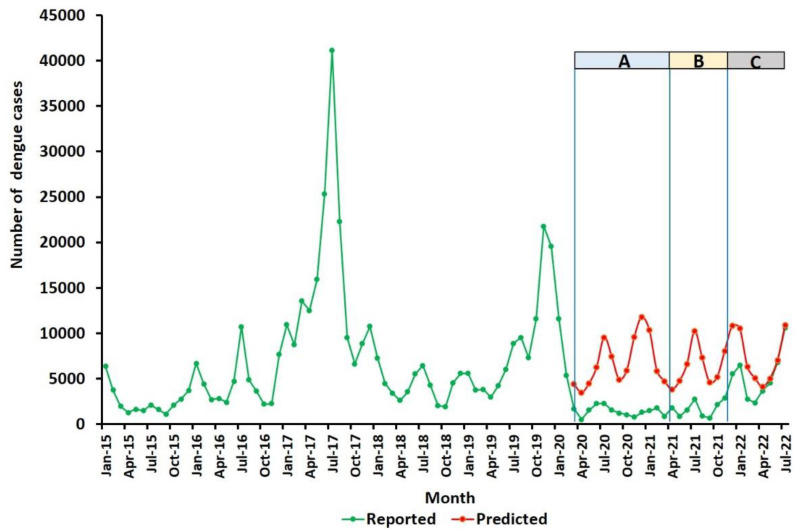
Country-wide predicted and actual numbers of dengue cases in Sri Lanka. Colour coding for periods A, B, and C is the same as in Table 1.

The numbers of dengue cases reported every month from 1 January 2015 to 31 July 2022 and the SARIMA model-predicted number of cases from 1 March 2020 to 31 July 2022 for Sri Lanka are graphically illustrated in Figure 2. The presented data include the previously reported findings from 1 March 2020 to 30 April 2021 (labelled as period A in Figure 2 and Table 1), when stringent movement restrictions were in place in response to COVID-19 [1]. During the period after 22 November 2021, when the movement restrictions were completely removed (period C in Figure 2), the actual number of dengue cases per month tended to approach the predicted number, which was particularly evident from April to July 2022 (Figure 2 and Table S1). 

### 3.2. Dengue Incidence from 1 May 2021 to 31 July 2022 in Each of the 25 Districts of Sri Lanka 

The total numbers of predicted and reported dengue cases during the 15-month period from 1 May 2021 to 31 July 2022 (periods B and C) for each of the 25 districts are shown in Supplementary Table S2. A comparison of the predicted and actual dengue cases for each month in each of the 25 districts shows that the actual number of reported dengue cases were significantly lower (*p* ≤ 0.05) than the predicted number of dengue cases in all but seven districts (Supplementary Table S3). These seven districts included the most populous districts of Colombo and Gampaha in the southwest wet zone of the island that receives rainfall from both the southwest monsoon (typically from April to June) and northeast monsoon (commonly from October to December). Similar analyses performed for the whole of period C from 1 December 2021 to 31 July 2022, when movement restrictions were removed, showed fewer districts with significant reductions in dengue cases (Table S3), which is consistent with the increasing convergence of predicted and actual numbers of dengue cases toward the end of this period (Figure 2 and Table S2). 

### 3.3. Dengue Incidence in Relation to Rainfall in the Jaffna District from 1 May 2021 to 31 July 2022

**Figure 3:**
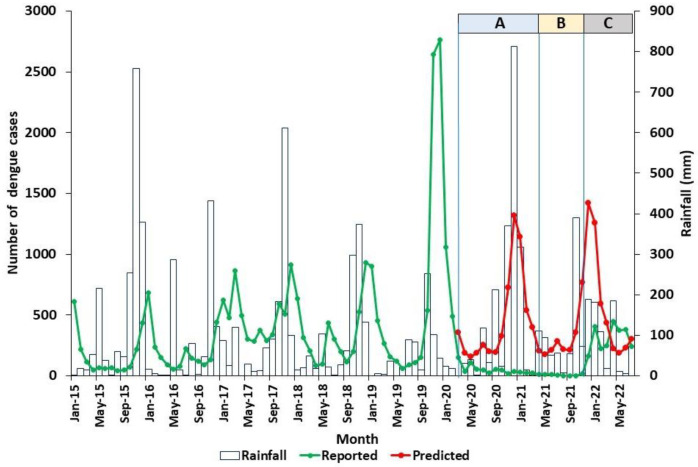
Predicted and actual numbers of dengue cases in relation to rainfall in the Jaffna district. Colour coding for periods A, B, and C is the same as in Table 1.

The monthly reported numbers of dengue cases and rainfall from 1 January 2015 to 31 July 2022, as well as the predicted number of dengue cases from 1 March 2020 to 31 July 2022, for the Jaffna district are graphically illustrated in Figure 3. Dengue incidence in Jaffna district, which is located in the dry zone of the country, increases soon after the onset of the northeast monsoon, which typically prevails from October to December [1,10,11]. The rainfall pattern in Jaffna district from May 2021 to July 2022 was similar to the same period in previous years (Figure 3). The total number of dengue cases reported during the period from 1 May 2021 to 31 July 2022 was 2540, compared with a predicted number of 6889, representing a significant reduction of 63% (*p* = 0.008). However, the actual and predicted numbers of dengue cases tended to converge during period C, with the reported cases exceeding the predicted number in April, May, and June 2022 (Table S2 and Figure 3). 

### 3.4. Aedes Larval Collections from Ovitraps from May 2021 to April 2022 in Gurunagar, Jaffna City

The *Aedes* larval collection data for the period from May 2021 to April 2022 are shown in Supplementary Table S4 and compared with previously published data for the pre-pandemic period of May 2019 to December 2019 [10] and period A of severe population movement restrictions from August 2020 to April 2021 [1]. Monthly collections yielded a total of 1713 and 1243 *Ae. aegypti* and *Ae. albopictus* vectors, respectively, during the period from May 2021 to April 2022. Observed changes in *Aedes* collections between movement restriction periods and their statistical significance are presented in Table 2. 

The findings show that the marked reductions in the numbers of *Aedes* larvae and the proportions of ovitraps that contained *Aedes* observed during the period A of severe movement restrictions tended to reverse and return to pre-pandemic levels when the movement restrictions were reduced and ultimately eliminated during periods B and C, respectively. Changes in association with population movement restrictions were more evident for the principal and more anthropophagic dengue vector, *Ae. aegypti*, than for the secondary and less anthropophagic vector, *Ae. albopictus*. 

## 4. Discussion

Our findings show that the decrease in numbers of dengue cases during the most severe COVID-19-related people movement restrictions imposed in 2020 and 2021 in Sri Lanka tended to reverse to expected levels when the restrictions were reduced in late 2021 and removed altogether on 22 November 2021. Monthly reported dengue cases reached approximately expected levels nationwide and in many individual districts, including the Jaffna district, from about April 2022 onward until the end of data collection in July 2022. This result supports our previous assertion [1] that movement of people outside residences in Sri Lanka facilitates the transmission of dengue, which was also consistent with dengue epidemiological data from the US with respect to the importance of house-to-house movement of people [14]. Sri Lanka has a large population of school-attending children, and the closure of schools from 1 May 2021 during this study period considerably influenced the movement of people. Schools were partially opened beginning 21 October 2021 and completely opened from 22 November 2021. School closures and other restrictions on the movement of people from 1 May 2021 until 22 November 2021 are therefore likely to have strongly contributed to the lower-than-predicted number of dengue infections during this period. The lag in resumption of normal levels of dengue transmission after movement restrictions were lifted on 22 November 2021 until about April 2022 is postulated to be mainly due to *Aedes* vector dynamics, as previously proposed [1] and further elaborated below. The relationship of dengue cases with COVID-19 stringency index [8] was not examined in detail in this study because this index also includes public health measures other than population movement restrictions, e.g., the use of face masks, which are not expected to affect dengue transmission. 

Our findings on dengue transmission during the COVID-19 movement restrictions are wholly consistent with findings from the Colombo district during 2020 and 2021 [3]. The populous Colombo district normally has the highest district-wise dengue incidence in the country [1,3,5,7,11]. The study in the Colombo district in 2020 and 2021 importantly showed that during this period, (i) the unusual decrease in dengue cases was not due to infections with varying DENV serotypes, (ii) the number of dengue cases was significantly and negatively correlated to the extent of school closures and tended to be negatively correlated with the stringency index, and (iii) the number of dengue cases was positively correlated with container and premise larval indices for *Ae. aegypti,* although this did not achieve statistical significance.

In the present study, we also examined *Ae. aegypti* and *Ae. albopictus* larval collections from ovitraps placed in a built-up area of Jaffna city in the Jaffna district. This was a continuation of an ovitrap-based study initiated during the 2019 pre-pandemic period [10] and continued during the early phase of the COVID-19 pandemic, when stringent population movement restrictions were in place [1]. The findings from the present study revealed that the significantly decreased larval collections during pandemic-related severe movement restrictions [1] compared with the pre-pandemic period [10], for *Ae. aegypti* and *Ae. albopictus,* tended to revert to pre-pandemic levels with the removal of population movement restrictions. 

Dynamics of disease transmission by vector mosquitoes is simply described for a non-immune population by the Ross–MacDonald equation [15,16] which can be usefully applied to dengue transmission in the present context. The equation relates the number of secondary infections generated from a single infected person (R_0_) to vector parameters as follows:

R_o_ = ma^2^αβp*^n^*/r[−log_e_ (*p*)]

m  =  ratio of the number of vector mosquitoes to the number of humans;
a  =  average number of human blood meals taken by a mosquito in a day;
α  =  probability of transmission of pathogen from an infected human to a biting mosquito;
β  =  probability of transmission of a pathogen from an infected mosquito to a non-immune human during feeding;
*p*  =  daily probability of survival of the mosquito vector;
*n*  =  duration in days from infection of a biting mosquito until the mosquito becomes capable of infecting humans after the pathogen undergoes obligatory development in the mosquito, also termed the extrinsic incubation period;
r  =  recovery rate in humans (inverse of the average duration of infectiousness in days).

The anthropophagic *Ae. aegypti* and partly anthropophagic *Ae. albopictus* vectors are daytime feeders [9,17] that are highly prevalent in premises of schools, hospitals, government offices, transport hubs, and factories in Sri Lanka [10,18]. The closure of schools and offices, as well other forms of restrictions on the movement of people outside of residences, can therefore be expected to reduce blood feeding by the two vectors, particularly *Ae. aegypti*, leading to a reduction in ‘a’ in the equation. Any reduction in blood meals will result in reduced oviposition and decreased vector densities, manifesting as reduced values of ‘m’ and ‘p’. Lower values for ‘a’, ‘m’, and ‘p’ will diminish R_0_, which will be particularly impacted by its exponential relationship with ‘a’ and ‘p’. R_0_ is directly related to the rate of dengue transmission and therefore the number of dengue cases in the population. Reduced oviposition as a result of decreased blood feeding is consistent with ovitrap findings from Jaffna city during periods of population movement restrictions reported in the present study, as well as in the preceding study [1], in addition to reduced larval indices reported in the Colombo district in 2020 and 2021 [3]. 

However, an increased incidence of dengue during the COVID-19 pandemic has been reported in some other locations, e.g., Singapore city [19]. The difference with Singapore was discussed in detail previously and essentially attributed to better vector control in public places compared to residences in Singapore [1]. A marked increase in *Aedes* larval indices was observed in Bengalaru city, Karnataka, India, during the COVID-19 pandemic and ascribed to the complete cessation of vector control activities [20]. Therefore, location-specific factors are important considerations for interpreting the effects of the COVID-19 pandemic and associated population movement restrictions on the incidence of dengue. 

The possibility that movement restrictions, lack of public transport, and fear of contracting COVID-19 are factors that may have contributed to decreased attendance of dengue patients at health centres was previously discussed [1]. The Sri Lankan population has, for many decades, rapidly sought help for mosquito-borne diseases from public health centres, where treatment is available free of charge [21,22]. The continued reduction in dengue cases during the period of partial movement restrictions, the documented inverse relationship between dengue incidence and school closures, and the fact that no limitations were imposed with respect to seeking medical help during even the most severe movement restrictions suggests that access to healthcare and reluctance to report dengue had, at most, a very limited impact on the reporting of dengue cases during the pandemic-related movement restrictions in Sri Lanka. 

## Figures and Tables

**Table 1:** **Timeline of population movement restrictions with the aim of suppressing COVID-19 transmission from 1 March 2020 to 31 July 2022 in Sri Lanka**.

Period	Movement Restrictions	Affected Districts	^a^ Reference Period Related to other Tables and Figures	^b^ Stringency Index Range
1-03-2020 to 30-04-2021	Documented in [1]	Documented in [1]	A	11–100
01–05-2021 to 21–10-2021	All schools and educational institutions closed nationwide	All districts	B	51–91
20-08-2021 to 01-10-2021	Nationwide stay-at-home order, no public transport, other essential services functioned with limited staff.	All districts		
20-08-2021 to 31-10-2021	Interprovincial travel restricted	All districts		
21-10-2021	Partial opening of schools (grades 1–5) with fewer than 200 students	All districts		
31-10-2021	Travel restrictions lifted	All districts		
08-11-2021	Partial opening of schools (grades 10–13)	All districts		
22-11-2021 onward	Schools and public places opened with no movement restrictions	All districts	C	54

^a^ Colors used for the three reference periods A, B and C correspond to the colors used for the same periods in other figures and tables in this article. ^b^ Stringency index of public health measures from [8] on a scale from 0 (least stringent) to 100 (most stringent). Other data from the Epidemiology Unit, Ministry of Health, Sri Lanka, COVID-19 updates (https://www.epid.gov.lk/web/index.php?option=com_content&view=article&id=225&lang=enn (accessed 15 August 2022)) and the Department of Government Information Sri Lanka [https://www.news.lk/news/covid-19?start=0 (accessed 12 August 2022)].

**Table 2:** **Comparison of *Aedes* collections from ovitraps during different movement restriction periods shown in Table 1**.

Comparison	Periods Compared	Species	Change and Probability (*p*)
Proportion of ovitraps with larvae out of all ovitraps placed. Chi square test	Period A vs. Pre-pandemic period	*Ae. aegypti*	Significantly reduced in period A χ^2^ = 121.93 *p* < 0.00001
		*Ae. albopictus*	Significantly reduced in period A χ^2^ = 27.32 *p* < 0.00001
	Periods B and C vs. Pre-pandemic period	*Ae. aegypti*	Significantly reduced in periods B and C χ^2^ = 29.01 *p* < 0.00001
		*Ae. albopictus*	Significantly reduced in periods B and C χ^2^ = 11.40 *p* = 0.00073
	Period A vs. Periods B and C	*Ae. aegypti*	Significantly reduced in period A χ^2^ = 51.85 *p* < 0.00001
		*Ae. albopictus*	Significantly reduced in period A χ^2^ = 5.43 *p* = 0.02
	Periods B and C	*Ae. aegypti* vs. *Ae. albopictus*	Significantly more ovitraps with *Ae. aegypti* χ^2^ = 10.58 *p* = 0.001
Numbers of larvae per ovitrap per month. Mann- Whitney U test	Pre-pandemic period vs. Period A	*Ae. aegypti*	Significantly more in pre-pandemic period *p* = 0.0003
		*Ae. albopictus*	Significantly more in pre-pandemic period *p* = 0.0048
	Pre-pandemic period vs. Periods B and C	*Ae. aegypti*	Significantly more in pre-pandemic period *p* = 0.0017
		*Ae. albopictus*	Not significantly different, *p* = 0.1556
	Period A vs. Periods B and C	*Ae. aegypti*	Significantly more in periods B and C *p* = 0.0002
		*Ae. albopictus*	Significantly more in periods B and C *p* = 0.0006
	Periods B and C	*Ae. aegypti* vs. *Ae. albopictus*	Tendency to be higher for *Ae. aegypti* *p* = 0.0561
Comparison of numbers of larvae collected per ovitrap	Period A vs. Pre-pandemic Period	*Ae. aegypti*	96% reduced in Period A
		*Ae. albopictus*	79% reduced in Period A
	Periods B & C vs. Pre-pandemic Period	*Ae. aegypti*	46% reduced in Periods B & C
		*Ae. albopictus*	29% reduced in Periods B & C
	Period A vs. Periods B & C	*Ae. aegypti*	93% reduced in Period A
		*Ae. albopictus*	70% reduced in Period A

Pre-pandemic period, when ovitraps collections were performed from March 2019 to December 2019 as previously reported [10]; period A of severe movement restriction from March 2020 to April 2021, when ovitraps collections were performed from August 2020 to April 2021, as previously reported [1]; period B of partial movement restriction from May 2021 to November 2021 and period C from December 2021 onward, when all movement restrictions were removed, during which ovitrap collections were performed from May 2021 to April 2022 in the present study.

## Data Availability

All data generated during this study are included in this published article and its Supplementary Files.

## Supplementary Materials

The following supporting information can be downloaded at: https://www.mdpi.com/article/10.3390/tropicalmed7100287/s1, Table S1: Monthly predicted number of dengue cases for the whole Sri Lanka from 1 May 2021 to 31 July 2022; Table S2: Monthly predicted and reported number of dengue cases of each of the 25 districts of Sri Lanka from 1 May 2021 to 31 July 2022; Table S3: Statistical comparison of the predicted and reported actual dengue cases in each district of Sri Lanka from 1 May 2021 to 31 July 2022 (Periods B and C) and separately from 1 December 2021 to 31 July 2022 (Period C); Table S4: Ovitrap collections of *Aedes aegypti* and *Aedes albopictus* larvae in Gurunagar, Jaffna. References [1,10] are cited in the supplementary materials.
