# Supplementary material for: Dengue Incidence and Aedes Vector Collections in Relation to COVID-19 Population Mobility Restrictions
Source: Trop Med Infect Dis. 2022 Oct 7;7(10):287. doi: 10.3390/tropicalmed7100287 (PMC9612376; doi:10.3390/tropicalmed7100287)
Supplement: Supplementary file 1 [file tropicalmed-07-00287-s001.zip › Table _S1.pdf]

Supplementary Table S1. Monthly predicted number of dengue cases for the whole Sri Lanka from 1 May 2021 to 31 July 2022

**Month**	**Dengue Incidence in all of Sri Lanka**	
	**Actual**	**Predicted**
May 2021	876	4748
June 2021	1586	6632
July 2021	2776	10209
August 2021	878	7310
September 2021	691	4583
October 2021	2175	5178
November 2021	2890	8061
December 2021	5528	10826
January 2022	6509	10511
February 2022	2773	6309
March 2022	2344	5055
April 2022	3645	4087
May 2022	4518	5034
June 2022	6813	7046
July 2022	10594	10880
**Total**	**54596**	**106468**
Probability of significant difference between actual and predicted cases P by the Wilcoxon signed-rank test is 0.00006		

Legend to Table S1. Periods B and C are shown in colours that correspond to Tables and Figure in the main text.
