# Supplementary material for: Dengue Incidence and Aedes Vector Collections in Relation to COVID-19 Population Mobility Restrictions
Source: Trop Med Infect Dis. 2022 Oct 7;7(10):287. doi: 10.3390/tropicalmed7100287 (PMC9612376; doi:10.3390/tropicalmed7100287)
Supplement: Supplementary file 1 [file tropicalmed-07-00287-s001.zip › Table_S2.pdf]

Supplementary Table S2: Monthly predicted and reported number of dengue cases of each of the 25 districts of Sri Lanka from 1 May 2021 to 31 July 2022

Month	Colombo		Gampaha		Kalutara		Kandy		Matale	
	Actual	Predicted	Actual	Predicted	Actual	Predicted	Actual	Predicted	Actual	Predicted
May-21	258	575	102	367	64	302	37	288	6	89
Jun-21	588	860	289	676	112	439	40	414	17	139
Jul-21	959	1392	527	1197	290	726	129	622	58	182
Aug-21	286	964	113	898	83	485	68	500	33	160
Sep-21	245	519	123	541	84	279	32	444	9	95
Oct-21	789	576	444	474	170	269	102	547	32	189
Nov-21	934	863	728	767	152	409	131	636	35	412
Dec-21	1337	1265	1186	923	441	517	225	680	39	446
Jan-22	1295	1128	1259	671	329	509	310	523	55	263
Feb-22	499	660	332	368	161	334	113	307	42	142
Mar-22	349	515	246	323	204	319	102	236	25	130
Apr-22	504	406	346	285	303	260	157	205	33	67
May-22	834	534	421	397	403	331	326	310	60	100
June-22	1810	807	708	692	459	475	652	463	207	159
July-22	2809	1305	1236	1243	701	789	1241	688	247	212
**Total**	**13496**	**12369**	**8060**	**9822**	**3956**	**6443**	**3665**	**6863**	**898**	**2785**

Month	N Eliya		Galle		Hambantota		Matara		Jaffna	
	Actual	Predicted	Actual	Predicted	Actual	Predicted	Actual	Predicted	Actual	Predicted
May-21	4	31	18	536	18	51	15	83	10	178
Jun-21	3	43	55	804	39	78	93	112	10	214
Jul-21	5	101	62	980	68	150	111	273	4	286
Aug-21	2	60	24	719	14	100	31	234	3	220
Sep-21	3	31	41	372	16	69	23	142	1	215
Oct-21	3	35	74	371	40	79	51	137	2	355
Nov-21	10	44	65	677	25	110	47	144	21	768
Dec-21	12	52	135	686	58	120	82	167	165	1419
Jan-22	25	55	299	654	104	149	92	234	405	1258
Feb-22	11	34	176	357	58	113	75	157	222	591
Mar-22	14	20	176	310	40	99	60	116	247	437
Apr-22	11	18	284	309	91	77	110	101	450	228
May-22	18	38	374	640	139	57	141	100	374	188
June-22	25	57	523	958	134	90	239	135	382	228
July-22	48	136	725	1196	406	172	346	330	244	304
**Total**	**194**	**755**	**3031**	**9569**	**1250**	**1514**	**1516**	**2465**	**2540**	**6889**

Month	Kilinochchi		Mannar		Vavuniya		Mulativu		Batticaloa	
	Actual	Predicted	Actual	Predicted	Actual	Predicted	Actual	Predicted	Actual	Predicted
May-21	0	14	0	14	3	50	2	12	91	480
Jun-21	1	17	1	9	2	58	0	3	50	404
Jul-21	1	29	3	8	3	60	0	16	26	338
Aug-21	1	19	1	6	0	58	0	13	8	192
Sep-21	1	21	0	4	0	62	0	2	3	120
Oct-21	0	24	3	17	3	90	1	20	22	169
Nov-21	1	40	9	25	6	265	1	42	25	312
Dec-21	13	54	354	94	12	302	1	57	84	685
Jan-22	24	69	110	100	21	160	12	71	106	851
Feb-22	8	50	25	34	12	104	5	24	85	779
Mar-22	10	68	5	16	6	71	5	13	100	700
Apr-22	13	53	6	13	5	21	8	4	196	602
May-22	16	35	3	14	4	69	2	11	234	534
June-22	12	40	14	10	5	95	7	6	136	450
July-22	10	47	8	9	12	92	6	20	109	376
**Total**	**111**	**580**	**542**	**373**	**94**	**1557**	**50**	**314**	**1275**	**6992**

Month	Ampara		Trincomalee		Kurunegala		Puttalam		Apura	
	Actual	Predicted	Actual	Predicted	Actual	Predicted	Actual	Predicted	Actual	Predicted
May-21	40	226	7	462	56	113	12	45	31	63
Jun-21	19	217	6	433	84	155	27	67	18	90
Jul-21	11	244	1	437	213	233	41	105	30	144
Aug-21	6	191	2	199	77	160	18	52	14	109
Sep-21	3	154	5	134	47	78	6	42	9	63
Oct-21	22	157	6	120	158	76	19	53	9	63
Nov-21	19	403	40	334	236	156	39	117	12	131
Dec-21	53	785	86	1027	471	170	207	126	27	162
Jan-22	227	1104	110	1416	640	200	435	115	59	178
Feb-22	81	601	107	1045	209	127	222	61	37	126
Mar-22	83	430	102	1092	118	96	111	37	30	83
Apr-22	148	303	278	925	108	78	107	29	26	67
May-22	231	269	240	633	113	104	112	39	20	65
June-22	387	259	90	595	254	144	139	58	33	92
July-22	159	297	38	601	423	215	204	92	62	151
**Total**	**1489**	**5640**	**1118**	**9453**	**3207**	**2105**	**1699**	**1038**	**417**	**1587**

Month	Polonnaruwa		Badulla		Moneragala		Ratnapura		Kegalle	
	Actual	Predicted	Actual	Predicted	Actual	Predicted	Actual	Predicted	Actual	Predicted
May-21	11	120	12	63	5	9	35	319	38	126
Jun-21	8	151	58	132	15	34	26	407	25	173
Jul-21	7	266	47	199	25	74	85	591	70	233
Aug-21	8	155	17	135	11	48	33	395	25	206
Sep-21	4	110	14	102	3	24	13	241	6	136
Oct-21	5	134	117	152	20	31	44	205	39	133
Nov-21	10	218	202	197	16	31	63	285	63	161
Dec-21	20	260	229	198	29	36	126	298	136	181
Jan-22	34	274	26	186	251	26	37	320	244	182
Feb-22	62	224	5	90	52	5	22	256	152	121
Mar-22	80	160	8	78	41	3	27	264	155	101
Apr-22	145	165	8	71	45	7	34	251	229	100
May-22	125	163	4	64	47	5	33	345	244	136
June-22	124	200	16	136	139	63	65	440	253	186
July-22	34	366	157	142	90	94	642	646	637	252
**Total**	**677**	**2966**	**920**	**1945**	**789**	**490**	**1285**	**5263**	**2316**	**2427**

Legend to Table S2. The colours used correspond to periods B and C in other Figures and Tables.
