# Supplementary material for: Dengue Incidence and Aedes Vector Collections in Relation to COVID-19 Population Mobility Restrictions
Source: Trop Med Infect Dis. 2022 Oct 7;7(10):287. doi: 10.3390/tropicalmed7100287 (PMC9612376; doi:10.3390/tropicalmed7100287)
Supplement: Supplementary file 1 [file tropicalmed-07-00287-s001.zip › Table_S3.pdf]

Supplementary Table S3: Statistical comparison of the predicted and reported actual dengue cases in each district of Sri Lanka from 1 May 2021 to 31 July 2022 (Periods B and C) and separately from 1 December 2021 to 31 July 2022 (Period C)

District	1 May 2021 to 31 July 2022			1 December 2021 to 31 July 2022		
	Actual	Predicted	‡P value	Actual	Predicted	‡P value
Colombo	13496	12369	0.9341	9437	6620	0.1484
Gampaha	8060	9822	0.1514	5734	4902	0.3828
Kalutara	3956	6443	0.0006104	3001	3534	0.07813
Kandy	3665	6863	0.02155	3126	3412	0.5469
Matale	898	2785	0.0008545	708	1519	0.1094
N Eliya	194	755	0.0007229	164	410	0.007813
Galle	3031	9569	6.104e-05	2692	5110	0.007813
Hambantota	1250	1514	0.125	1030	877	1.000
Matara	1516	2465	0.01508	1145	1340	0.5469
Jaffna	2540	6889	0.008362	2489	4653	0.3125
Kilinochchi	111	580	0.0007247	106	416	0.007813
Mannar	542	373	0.06068	525	290	0.726
Vavuniya	94	1557	0.0007247	77	914	0.01415
Mullaitivu	50	314	0.001961	46	206	0.03906
Batticaloa	1275	6992	6.104e-05	1050	4977	0.007813
Ampara	1489	5640	0.0001831	1369	4048	0.02344
Trincomalee	1118	9453	6.104e-05	1051	7334	0.007813
Kurunegala	3207	2105	0.1054	2336	1134	0.007813
Puttalam	1699	1038	0.09933	1537	557	0.01415
Apura	417	1587	0.0007211	294	924	0.01415
Polonnaruwa	677	2966	0.0007247	624	1812	0.01415
Badulla	920	1945	0.0008545	453	965	0.03906
Moneragala	789	490	0.5508	694	239	0.04206
Ratnapura	1285	5263	6.104e-05	986	2820	0.007813
Kegalle	2316	2427	0.4887	2050	1259	0.02344

‡Probability P calculated by the Wilcoxon signed-rank test
