# Supplementary material for: Dengue Incidence and Aedes Vector Collections in Relation to COVID-19 Population Mobility Restrictions
Source: Trop Med Infect Dis. 2022 Oct 7;7(10):287. doi: 10.3390/tropicalmed7100287 (PMC9612376; doi:10.3390/tropicalmed7100287)
Supplement: Supplementary file 1 [file tropicalmed-07-00287-s001.zip › Table_S4.pdf]

Supplementary Table S4: Ovitrap collections of *Aedes aegypti* and *Aedes albopictus* larvae in Gurunagar, Jaffna

(i) Present Study May 2021 to April 2022 (periods B and C)

Month	*Aedes aegypti*		*Aedes albopictus*	
	No. of larvae	+ ve	No. of larvae	+ ve
May 2021	121	5/10	53	2/10
June 2021	164	6/10	71	3/10
July 2021	160	6/10	68	2/10
August 2021	202	7/10	65	2/10
September 2021	212	8/10	81	3/10
October 2021	198	6/10	88	3/10
November 2021	153	6/10	96	3/10
December 2021	165	5/10	115	3/10
January 2022	106	4/10	176	5/10
February 2022	95	5/10	119	4/10
March 2022	105	5/10	146	4/10
April 2022	32	2/10	165	6/10
**Total**	**1713**		**1243**	

(ii) August 2020 to April 2021 - data from [1]

Month	*Aedes aegypti*		*Aedes albopictus*	
	No. of larvae	+ ve	No. of larvae	+ ve
August 2020	22	2/10	18	2/10
September 2020	56	3/10	40	2/10
October 2020	12	1/10	0	0/10
November 2020	0	0/10	56	3/10
December 2020	0	0/10	18	1/10
January 2021	0	0/10	70	4/10
February 2021	0	0/10	6	1/10
March 2021	0	0/10	16	1/10
April 2021	0	0/10	58	3/10
**Total**	**90**		**282**	

(iii) March 2019 to December 2019 - pre-pandemic data from [10]

Month	*Aedes aegypti*		*Aedes albopictus*	
	No. of larvae	+ ve	No. of larvae	+ ve
March 2019	109	6/9	7	1/9
April 2019	289	9/9	117	4/9
May 2019	285	9/9	132	7/9
June 2019	246	7/9	109	6/9
July 2019	235	9/9	170	6/9
August 2019	261	8/9	160	5/9
September 2019	274	9/9	205	6/9
October 2019	281	9/9	181	6/9
November 2019	199	8/9	184	5/9
December 2019	201	6/9	55	5/9
**Total**	**2380**		**1320**	

Legend to Table S4. **+ve** – number of ovitraps with *Aedes* larvae out of the total number of ovitraps placed. Colour codes correspond to periods A, B and C in text and Table 1.
